# Application of Coal Gangue as a Coarse Aggregate in Green Concrete Production: A Review

**DOI:** 10.3390/ma14226803

**Published:** 2021-11-11

**Authors:** Shan Gao, Sumei Zhang, Lanhui Guo

**Affiliations:** 1 School of Civil Engineering, Harbin Institute of Technology, Harbin 150090, China; smzhang@hit.edu.cn (S.Z.); guolanhui@hit.edu.cn (L.G.); 2 Shaanxi Key Laboratory of Safety and Durability of Concrete Structures, Xijing University, Xi’an 710123, China

**Keywords:** coal gangue, green concrete, coarse aggregate, structural member, eco-friendly production

## Abstract

Among the techniques for converting stacked coal gangue to reusable material, one of the most effective ways is to use coal gangue as a coarse aggregate in green concrete productions. The physical and chemical properties of rock and spontaneous-combustion coal gangue are generally suitable for being used as a coarse aggregate in green concrete. Coal gangue concrete is not recommended to be used in subsurface structures, as its water absorption law would be changed under a large replacement ratio. The mechanical performance of coal gangue concrete is degraded by raising the replacement ratio. Over-low and -high concrete grades are not suggested to be used as coal gangue aggregate, unless extra admixtures or specific methods are used. The influence of coal gangue on the durability of coal gangue concrete is remarkable, resulting from the porous structure of coal gangue that provides more transmission channels for air and liquid in concrete, but is beneficial for thermal insulation. The usage of coal gangue in structural concrete members is still limited. The mechanical behavior of some structural members using coal gangue concrete has been reported. Among them, concrete filled steel tubes are a preferable configuration for using coal gangue concrete, regarding both the mechanical and durability performance.

## 1. Introduction

**Figure 1:**
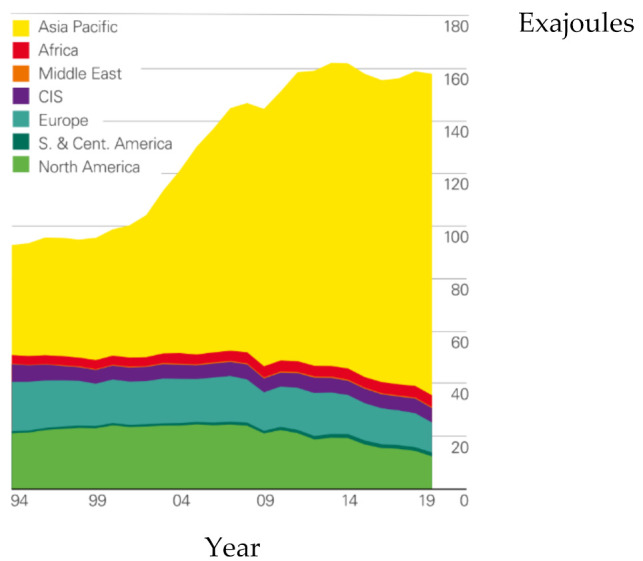
Coal consumption by region [1].

Even though many kinds of new energy and renewable fuels have been explored nowadays, coal is still the main energy for many countries in the world, especially for countries in Asia Pacific region, such as China, Indonesia, and Vietnam. In fact, the increase of global coal production in 2019 resulted from the significant production increases of China and Vietnam, while that of other regions in the world declined, as shown in Figure 1 [1]. Coal still accounts for more than 70% of energy expenditure in China [2], while the world coal expenditure fell to its lowest level in 16 years, as a result of being displaced by natural gas and renewables.

Along with the excavation and washing of coal in the production process, large amounts of rock-like coal gangue (CG) accompanying the carboniferous beds are discharged as industrial residues, accounting for around 10–20% of the coal production [2,3,4]. In the largest coal production country, namely China, more than 700 million tons of coal gangue was expected in 2020, with the annual rate of 300–350 million tons [4,5], not to mention that almost 5 billion tons of that have been accumulated in China [4,6] and the number for Europe is 175 million tons [2].

Even though the storage location of coal gangue is normally far away from downtown areas, the considerable stockpile of coal gangue still threatens human society, besides the natural ecosystem. Firstly, from the point of geotechnical engineering, rock-like coal gangue has to be stored in dumps, which obviously occupy enormous land resources that can be used for farming and construction. Furthermore, normal disordered gangue stacking tends to result in debris flow [2,4,5]. Secondly, it is well-known that the composition of coal gangue changes along with storage time and environment [7]. In other words, even though the major chemical compositions of gangue are SiO_2_ and Al_2_O_3_ in the form of quartz and feldspar [3], certain traces of various heavy metal elements, including Pb^2+^, Zn^2+^, and Cu^2+^, are still contained in coal gangue. After long-term weathering and soaking, the leaching of heavy metals would result in the contamination of soil, groundwater, and surface water, further harming the surrounding ecosystem and human health through bioaccumulation [2,4,6,7]. Last but not least, stocked coal gangue is also an emission resource of volatile organic chemical gas. The heat continuously accumulating inside the stocked coal gangue leads to the spontaneous combustion of coal gangue in the form of fire, even blast disasters, due to the existence of unburned coal, pyrite, and oxygen, releasing a large amount of harmful gases, including SO_2_, NO_x_, and CO, into the atmosphere. It is a pity that this air pollution from coal gangue is hard to control [2,4].

The gangue utilization rate of the main developed regions, such as US and Europe, is more than 90%. In contrast, that value in the largest coal gangue production country, namely China, is only about 60–70%, while the coal gangue utilization of China through combustion and calcination normally results in secondary pollution [4,6]. How to deal with the accumulation of coal gangue and improve its utilization rate has become one of the most crucial issues related to the sustainable development of the coal industry.

## 2. Utilization of Coal Gangue

For years, researchers have been working on the effective utilization of the enormous stocked coal gangue. In China, more than a half of discharged coal gangue is used for land reclamation—one third of that is used for power generation and the rest is used for producing building materials [2,4].

However, the main compositions of natural rock coal gangue are inactive SiO_2_ and Al_2_O_3_, whose cementitious ability is rather weak. Calcination or spontaneous combustion are necessary to add CaO to coal gangue. For now, the percentage of calcined gangue combination with Portland cement is still low, normally less than 15% [3,6]. In contrast, as additive and fine aggregates, calcined gangue could also be used to prepare cementitious material, and is a promising replacement for fly ash and slag [8,9]. Meanwhile, coal gangue can be crashed and directly used as a lightweight aggregate, with a good internal curing capacity, but a high demand for water for mixing [6,10,11].

Besides power generation, the current utilization methods of coal gangue, including land reclamation and producing cementitious materials, tend to cause secondary environmental contamination and extra energy consumption, as the calcination and superfine grinding of coal gangue seem inevitable. In contrast, the utilization of coal gangue as a coarse aggregate has a relatively low energy consumption and environmental impact. However, currently, most of the studies on the utilization of coal gangue focus on producing cementitious materials and additives. The limited studies on the utilization of coal gangue as a coarse aggregate only consider the mechanical and durability performance of the concrete material. The structural application of coal gangue coarse aggregate concrete, such as in beams, columns, and walls, is rather little and is not deep enough, which also includes design methods for structural members with coal gangue concrete.

Hereafter, this paper introduces the direct utilization of coal gangue for preparing structural concrete as a replacement of coarse aggregates, aiming at sustainable, comprehensive, and efficient cleaner production. Through this review, it is hoped that more structural applications of coal gangue coarse aggregate concrete would be proposed and studied experimentally and theoretically.

## 3. Methods

The references for the review were searched for in December 2020 in Scopus and the China National Knowledge Internet database, with the consideration of the following factors [4]:

(1) The presence of terms “coal gangue” and “coarse aggregate” in the documents;
(2) The considered period: 1990–2020;
(3) The publication form of paper: “Article” or “Review”;
(4) Searching area: “Engineering” and “Material”.

**Figure 2:**
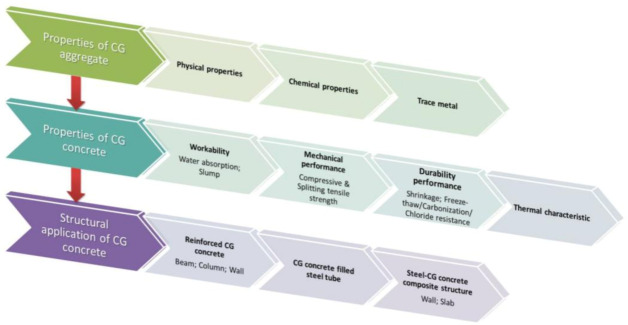
Flowchart of the review.

It should be mentioned that as the focus of this review is the coal gangue utilization of coarse aggregates, most of the references regarding gangue utilization in producing cementitious materials are not mentioned. In fact, studies on gangue utilization of coarse aggregates for the preparation of concrete mainly performed by Chinese researchers are much less than that for the production of cementitious materials, as China remains the largest production country in the world and the coal consumption in other countries and regions has been dropping for decades. Referring to the application of coal gangue in concrete structural members, the relevant studies have only been performed by Chinese researchers for the same reason. The main work of this review is summarized in Figure 2.

## 4. Properties of Coal Gangue Aggregate

### 4.1. Physical Properties

Natural coal gangue (namely rock coal gangue) is a rock-like mining waste that normally appears from gray to black. The color changes to whitish gray and even red after spontaneous combustion or after being calcinated, according to its iron oxide content and weathering degree [2]. According to Chinese standard JGJ-52-2006, the physical properties of rock coal gangue (RCG) and spontaneous-combustion coal gangue (SCG) vary from different origins in China, as summarized in Table 1 [12,13,14,15,16,17,18,19,20,21,22,23,24]. 

The apparent density of gangue is approximately 2600 kg/m^3^, which is similar to natural stone, while its bulk density is approximately 1200 kg/m^3^, which belongs to light-weight aggregate. The water absorption and crushing value of gangue (approximately 5% and 20% respectively) are relatively larger than those of natural stone (approximately 2% and 15%, respectively) due to its higher carbon content. Although calcination would reduce the carbon content in RCG, its water absorption and crushing value would also be further enlarged. Even though some crashing values of CG in Table 1 are close to or even smaller than the conventional crashing values of natural rock, these values are still larger than those of the natural rock collected from the local in the corresponding references.

As the replacement of natural coarse aggregate, both RCG and SCG seem to be a little fragile to be used in concrete, which may likely result in the fracture in coal gangue aggregate itself, besides the fracture in the interfacial transition zone (ITZ).

### 4.2. Chemical Properties

According to its chemical properties, coal gangue could be categorized into four types, namely claystone gangue (SiO_2_: 40–70% and Al_2_O_3_: 15–30%), sandstone gangue (SiO_2_ > 70%), aluminous stone gangue (Al_2_O_3_ > 40%), and calcareous stone gangue (CaO > 30%) [25]. Table 2 lists the mineralogical composition of coal gangue in various countries [26]. Typical coal gangue contains 50–70% clay minerals, 20–30% quartz, and 10–20% other minerals and carbon impurity. Compared with Europe and South America, the content of kaolinite in the coal gangue of China and USSR is richer, showing more potential for reuse, as kaolinite is apt to be activated. Instead, the content of illite in the coal gangue of Europe is much richer than that in USSR and China.

In general, as coal gangue is actually a transition substance between rock and coal, its chemical composition is similar to that of natural coarse aggregate, which also confirms the feasibility of being a coarse aggregate in concrete production.

As listed in Table 3, the content of SiO_2_ is always maximum among the chemical composition in coal gangue, regardless of the origins, while the content of Al_2_O_3_ is normally half that of SiO_2_. The major chemical compositions of coal gangue, namely SiO_2_ and Al_2_O_3_, tend to increase after spontaneous-combustion or being calcinated, but still vary from different origins. As the content of (SiO_2_ + Al_2_O_3_ + Fe_2_O_3_) in gangue normally accounts for more than 70%, coal gangue could be treated as a pozzolanic material, regardless of its origin [2].

### 4.3. Trace Metal

The heavy metal content of coal gangue and the permissible values in soils and surface lands are listed in Table 4. Similar to the chemical composition, the heavy metals in gangue also vary according to the origins. The Cr content of coal gangue in Italy is much higher than that in China and Poland, while the As content of coal gangue in China is a little higher than the permissible value. Besides this, the heavy metal content of gangue is much lower than the permission level of the Polish Regulation of the Ministry of Environment of 9 September 2002 for soil quality standards and earth quality standards. In addition, the short-term leachability of the heavy metals is relatively weak [5]. In this case, in terms of environmental protection, coal gangue could also be used as a coarse aggregate in green concrete production. However, more data regarding the heavy metal content in coal gangue should be collected from testing in order to propose a more accurate requirement regarding the heavy metal content of discharged coal gangue. In addition, the content of heavy metal in discharged coal gangue should be tested before being used in a concrete mixture so as to make sure that its utilization would not pose an environmental, health, and safety concern.

## 5. Properties of Coal Gangue Concrete

Even though using crashed and sieved coal gangue as a coarse aggregate is the most convenient and energy-saving method to utilize coal gangue, the feasibility of this method still depends on the properties of coal gangue. The performance of coal gangue concrete needs to meet the standard for preparing the concrete in construction. Calcination is beneficial for improving the interfacial bond between coal gangue and cement mortar.

### 5.1. Workability

#### 5.1.1. Water Absorption

**Figure 3:**
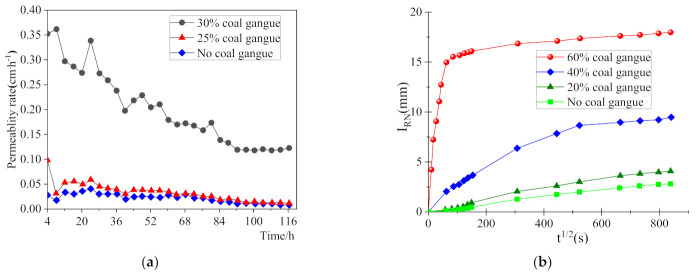
Effect of rock coal gangue replacement on the water absorption of coal gangue concrete. (**a**) Permeability coefficient [31]; (**b**) Absorption depth [32].

It is foreseeable that the water absorption of coal gangue concrete would be increased by raising the coal gangue replacement ratio, as its water absorption is higher than that of natural gravel. Figure 3a shows the permeability coefficient of the concrete under 25% and 30% replacement ratios. The increase of the pore phase conductivity by increasing the coal gangue replacement is supposed to result in an increase of the permeability coefficient [31]. Furthermore, the water absorption law tested according to ASTM C1585 was remarkably changed, as shown in Figure 3b. The inflection point for the water absorption speed in the curves became more and more obvious with the increase of the replacement ratio. Under a large replacement ratio, coal gangue aggregate in concrete became almost saturated in a short time, and then the unsaturated mortar and natural aggregates continued to absorb water, with a water absorption speed much lower than that of the coal gangue. Comparing the results in [31,32], as shown in Figure 3b, it seems a coal gangue replacement ratio lower than 30% would not significantly affect the water absorption of the concrete. In general, coal gangue concrete is not recommended for use in subsurface structures; besides, it is obvious that a large water absorption would reduce the strength of the coal gangue concrete.

#### 5.1.2. Slump

**Figure 4:**
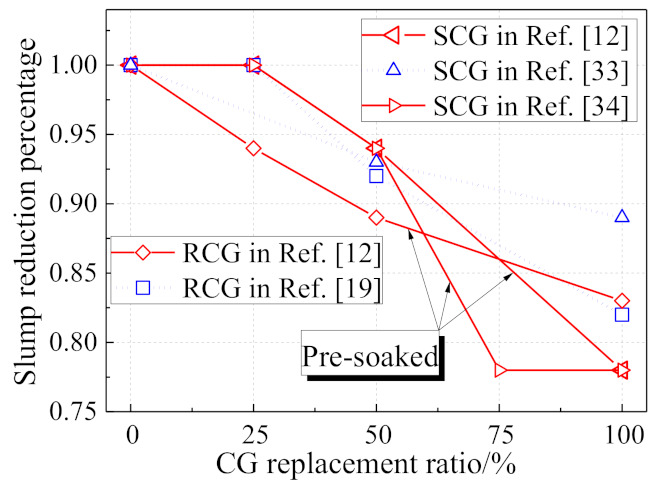
Effect of coal gangue replacement on the slump of coal gangue concrete.

**Figure 5:**
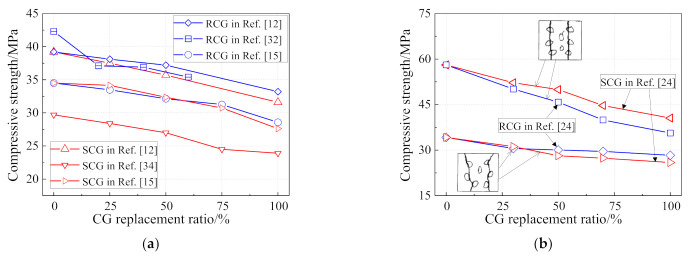
Effect of coal gangue replacement on the compressive strength of coal gangue concrete. (**a**) Compressive strength; (**b**) Failure pattern.

As coal gangue (CG) shows a larger water absorption capacity than natural aggregate, the workability of coal gangue concrete would obviously be decreased by raising the CG replacement ratio according to Chinese standard GB 50164-2011, as shown in Figure 4. Under a 50% CG replacement ratio, the slump of the coal gangue concrete would be reduced by about 10%, while the number would be 20% under 100% CG replacement ratio. As the properties of CG vary from the origin, the difference between RCG and SCG shown in Figure 5 is subtle [33,34]. To enhance its workability, adding fly ash and superplasticizer in coal gangue concrete are both effective and simple methods [35,36]. Additional water consumption for coal gangue could also be a choice to enhance the workability, but would result in a strength reduction of coal gangue concrete resulting from the extra water [37]. It is surprising from Figure 4 that the pre-soaking method is not as effective as it is supposed to be, which may be also due to the properties of coal gangue varying from the origin.

### 5.2. Mechanical Performance

#### 5.2.1. Compressive Strength

It is assumed that the porous structure of CG results in a strength reduction of coal gangue concrete. As shown in Figure 5a, the compressive strength of coal gangue concrete tested according to Chinese standard GB 50010-2010 decreases approximately linearly with the rise of the replacement ratio, regardless of concrete strength and the type of coal gangue. Under 100% CG replacement ratio, the compressive strength of coal gangue concrete decreased by around 15–20% [12,15,32,34]. Figure 5b indicates that the failure pattern of coal gangue concrete is different according to the concrete grade [24]. Under a low concrete grade, the failure of coal gangue concrete occurs in the mortar, where the coal gangue shows little effect. In contrast, under a high concrete grade, the failure of coal gangue concrete happens in both the mortar and coal gangue aggregate. In this case, the types of coal gangue aggregate contribute to the compressive strength of coal gangue concrete. As mentioned above, calcinating CG would activate the cementitious composition in it and improve the strength of the mortar, but would degrade the strength of the coal gangue itself. In addition, an over-low concrete grade, for example lower than C20, is not suggested for use as a coal gangue aggregate, as the mortar is not enough to fully cover the coarse aggregate, which may lead to a significant reduction in the mechanical properties of coal gangue concrete [38].

Similar to ordinary concrete, adding steel fiber could reinforce the mechanical properties of coal gangue concrete. The compressive strength of coal gangue concrete with 2% steel fiber would be improved by 16.7% [39]. Furthermore, calcianting RCG [40] and presoaking SCG [17] would also achieve the same effect.

**Figure 6:**
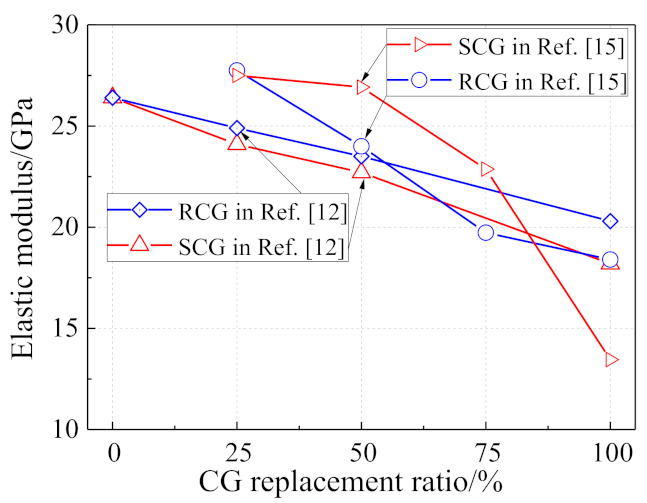
Effect of coal gangue replacement on the elastic modulus of coal gangue concrete.

As presented in Figure 6, even though a general descending trend in the elastic modulus of coal gangue concrete was found when raising the CG replacement ratio, the comparsion between the RCG concrete and SCG concrete was contrary between [12] and [15]. In other words, the elastic modulus of RCG concrete was larger than that of the SCG concrete [12], whiet the former was smaller than the latter in [15], except for the 100% replacement ratio. Besides the discreetness of coal gangue properties, the pre-soaking method, which is supposed to effectvely improve the mechanical behaviors of SCG concrete, rather than those of RCG concrete, was used in [15].

**Figure 7:**
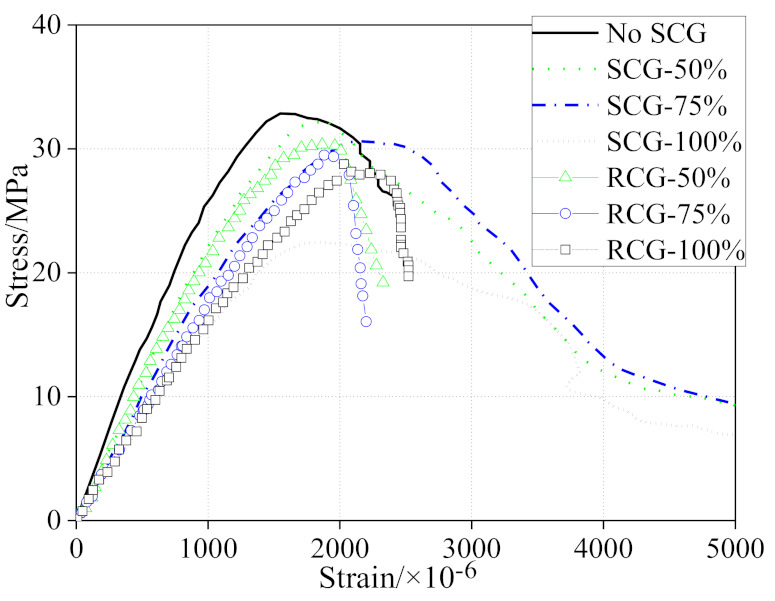
Stress−strain curves of the coal gangue concrete [15].

Figure 7 presents the stress−strain relationship of the concrete with different contents of RCG and SCG [15]. It indicates that increasing both the RCG and SCG replacement ratios would increase the peak strain of the coal gangue concrete. The drop beyond the peak in the curves of RCG concrete was sharper than that of the SCG concrete, due to the porous structure of SCG. These phenomena imply that the ductility of SCG concrete is better than that of RCG concrete. Based on the tested stress−strain relationship of coal gangue concrete and the model in Chinese standard GB 50010-2010, a dimensionless model (*x* = *ε*/*ε_c_*, *y* = *σ*/*σ_c_*) for both RCG and SCG concrete is proposed for the first time, as follows [15]. The proposed model should consider the aggregate replacement ratio and CG types. However, more data regarding the stress−strain relationship model of CG concrete are still needed to validate the model, as it was empirically obtained and the properties of CG vary according to the origin.

(1) $$y=\left\{\begin{array}{c} ax+(3-2a)x^{2}+(a-2)x^{3},0\leq x\leq 1\\ x/(b{\left(x-1\right)}^{2}+x),x>1 \end{array}\right.$$

where *a* is (−0.0112*r* + 1.844) and (7 × 10*r*^−5^ − 0.0117*r*^2^ + 1.6866) for RCG and SCG, respectively, and *b* is (0.1052*r* + 1.824) and (−0.0118*r* + 2.38) for RCG and SCG, respectively.

#### 5.2.2. Splitting Tensile Strength

**Figure 8:**
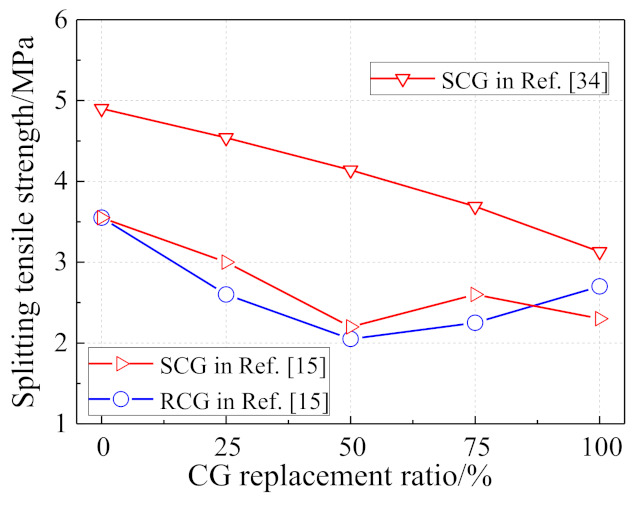
Effect of coal gangue replacement on splitting tensile strength of coal gangue concrete.

As shown in Figure 8, generally, the splitting tensile strength of coal gangue concrete decreases as the CG replacement ratio increases. However, due to the discreetness of thecoal gangue properties, a minimum value was observed in the results of RCG concrete in [15] under 50% CG replacement ratio. By using CG as the coarse aggregate, more splitting tensile damage was found in the coarse aggregate, namely coal gangue, rather than in the bond between the coarse aggregate and the matrix.

### 5.3. Durability Performance

#### 5.3.1. Shrinkage

**Figure 9:**
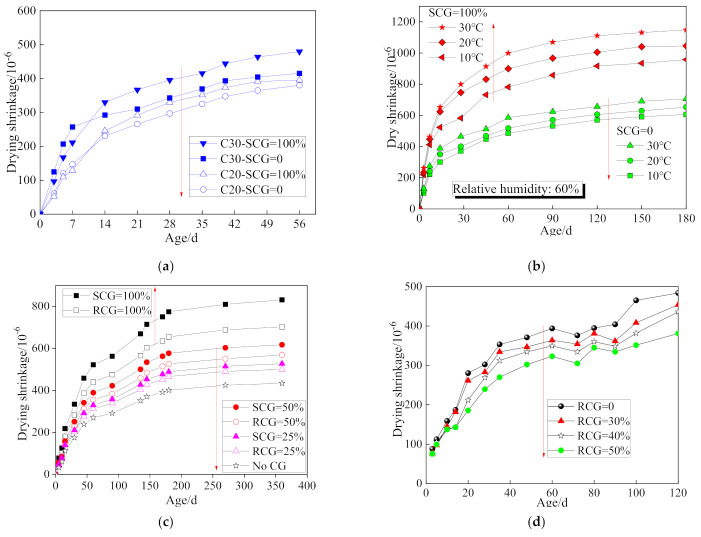
Effect of coal gangue replacement on the shrinkage of coal gangue concrete. (**a**) Concrete grade [41]; (**b**) Curing condition [42]; (**c**) Coal gangue type [12]; (**d**) Replacement ratio (RCG) [43].

The cracks resulting from shrinkage affect the durability of concrete, especially for concrete with CG whose water absorption characteristic is different to the natural coarse aggregate. Figure 9a shows the effect of concrete grade on the coal gangue concrete shrinkage tested according to the GB/T 50082-2009 standard and ASTM C157. It is obvious that higher-grade coal gangue concrete shows larger shrinkage. It is also interesting that the early shrinkage of coal gangue concrete is smaller than that of normal concrete, which results from the inner curing effect of coal gangue with a large water absorption [41]. As shown in Figure 9b, the increase of shrinkage due to the increase of curing temperature would be enlarged by raising the CG replacement ratio [42]. As expected, the concrete using an SCG aggregate and large replacement ratio showed large shrinkage, as shown in Figure 9c [12]. However, a totally opposite trend, where increasing the replacement ratio decreased the shrinkage of concrete was still observed, as seen in Figure 9d [43]. This finding may result from the larger volume of coal gangue used in the concrete mixture under the same mass replacement ratio of the coarse aggregate, which would reduce the shrinkage of the mortar matrix.

#### 5.3.2. Freeze−Thaw Resistance

The failure of concrete under freezing−thawing cycles results from the frost−heave stress related to the capillary water absorption properties [44]. Due to the large frost−heave stress resulting from the porous structure of CG, the mechanical performance of coal gangue concrete was significantly affected by freezing−thawing cycles from the test complying with Chinese standard GB/T 50082-2009, which was enlarged by raising the CG replacement ratio [32,45]. The freezing−thawing mass loss of coal gangue concrete would also be enlarged by increasing the water−cement ratio [46].

Based on the tested results, a dimensionless model (*x* = *ε*/*ε_c_*, *y* = *σ*/*σ_c_*) of the stress−strain relationship is proposed for coal gangue concrete after freezing−thawing cycles [23]. The model modified from the model in Chinese standard GB 50010-2010 considers the effects of the CG replacement ratio and the cycle number of freezing−thawing, rather than the CG type. Therefore, similar to the model proposed in [15], even though this model fit well with the test results, it was also empirically obtained, which means that more data are needed in order to validate it.

(2) $$y=\left\{\begin{array}{c} (1.92-\mu N)x+(1.08+\mu N)x^{2}-(0.08+\mu N)x^{3},0\leq x<1\\ x/((1.3+\eta N){\left(x-1\right)}^{2}+x),x\geq 1 \end{array}\right.$$

where *μ* is (0.0025*e*^0.0552*r*^*^CG^*), *η* is (0.0035 + 0.0011*r*_CG_), *N* is the cycle number of freezing−thawing, and *r*_CG_ is the CG replacement ratio.

The admixture of fly ash and silica fume [47], organic anti-freezing agent [48], and polypropylene fiber [49] are added into the coal gangue concrete to enhance its freeze−thawing resistance. Up to 1% of organic anti-freezing agent, namely ethylene glycol, could improve the freeze−thawing resistance of coal gangue concrete by 10–30%, which is believed to be one of the most effective methods.

#### 5.3.3. Carbonization Resistance

**Figure 10:**
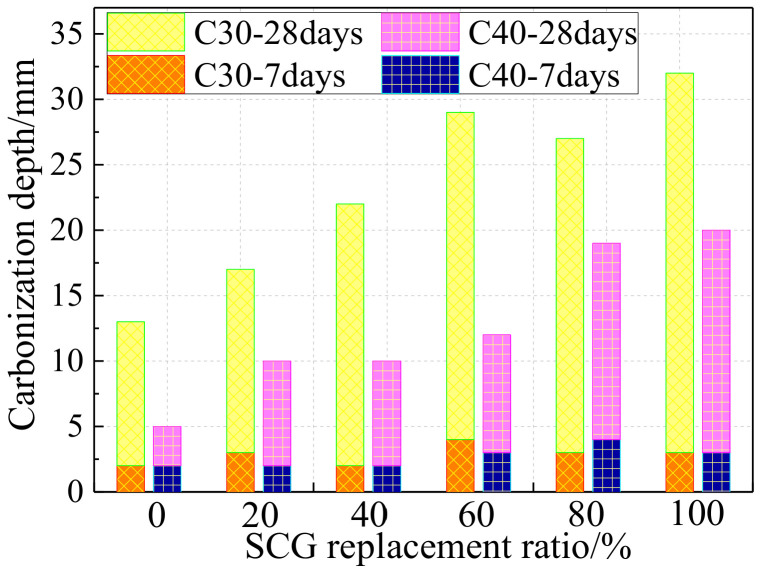
Carbonization depth−SCG replacement ratio relationship.

The transmission of CO_2_ in concrete is mainly affected by the porosity ratio and connected channels. The coal gangue aggregate generally has more pores than natural gravel. The air containing CO_2_ enters into the concrete more easily, resulting in a poor durability of the concrete [50]. Therefore, it is obvious that increasing the coal gangue replacement ratio would reduce the carbonization resistance of coal gangue concrete (seen in Figure 10, showing the results from the test complying with Chinese standard GB/T 50082-2009) [51].

Figure 10 also implies a finding that the carbonization depth of SCG concrete is negatively related to its strength, which was also found in other relavent tests [52,53]. A formula describing the relationship between carbonization depth *d*_c_ and strength *f*_CG_ of SCG concrete after 28 days of curing is proposed as follows [53]. This empirical model is not only related to one parameter, namely CG strength, but is also valid in the scope of concrete strength.

(3) $$d_{c}=-0.31f_{CG}+29.6,(18.7\leq f_{c}\leq 51.2)$$

Compared with RCG, the concrete using 700 °C-calcinated coal gangue has a better carbonization resistance than that of RCG concrete. C-S-H hydrogel, which is produced by the second hydration reaction among the SiO_2_, Al_2_O_3_, and cement hydration product Ca(OH)_2_ produced after the calcination of the gangue aggregate, compacts the pores and reduces the CO_2_ transmission channel in concrete. A formula for predicting the carbonization depth of RCG concrete regarding age (*t*), water−cement ratio (*m*_w_/*m*_c_), and coal gangue replacement ratio (*r*_CG_) is proposed [54]. Compared with Equation (3) from [53], this empirical formula considers the effects of three different parameters, instead of just the concrete strength. However, as mentioned by the authors themselves, this formula is only suitable for RCG, rather than SCG.

(4) $$d_{c}=0.098\sqrt{t}+50.66m_{w}/m_{c}+2.015r_{CG}-17$$

#### 5.3.4. Chloride Resistance

More capillary pores in SCG are the main reason for the poor chloride resistance of coal gangue concrete. Therefore, in the coal gangue concrete with a low water−cement ratio, the gel pores and capillary pores are filled with hydrated gel, increasing the compactness of the concrete larger, decreasing the electric flux, and enhancing the chloride ion penetration resistance [55,56,57].

**Figure 11:**
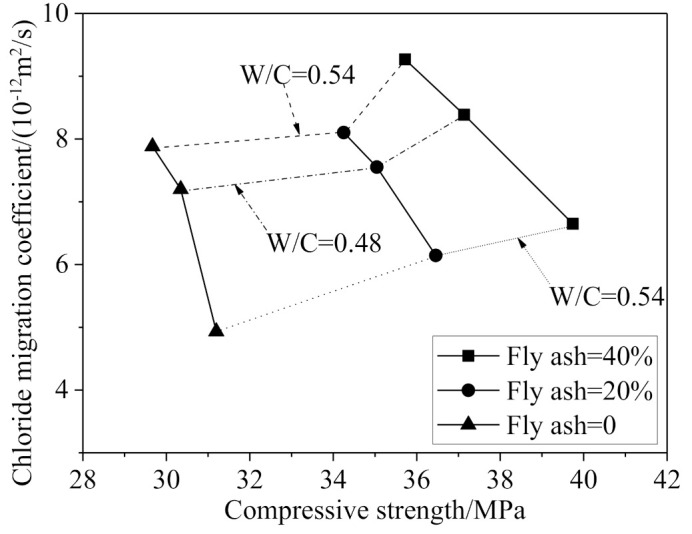
Relationship between chloride migration coefficient and strength of coal gangue concrete [57].

To improve the chloride resistance of coal gangue concrete, calcination [24], adding steel fiber [39], and adding the mineral admixture and superplasticizer [58] are effective ways that could reduce the micro cracks of mortar matrix and change the ion permeation path. As shown in Figure 11, where the results are from the test complying with ASTM C 1202-05, adding fly ash would reduce the strength of the coal gangue concrete, but increase its chloride resistance (namely decreasing the chloride migration coefficient), regardless of the water−cement ratio [57].

### 5.4. Thermal Characteristic

**Figure 12:**
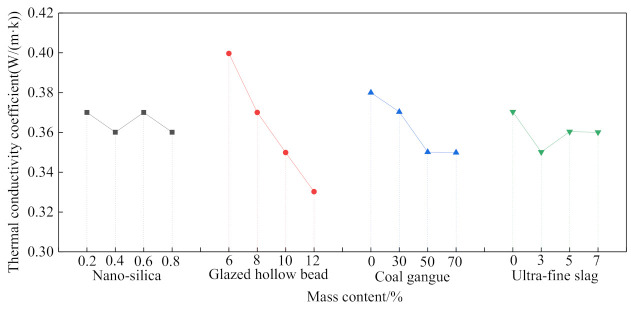
Thermal conductivity coefficient of the novel coal gangue concrete [19].

As mentioned above, increasing the CG content would normally degrade the mechanical and durability performance of the coal gangue concrete. In contrast, the porous structure of CG is beneficial for thermal insulation. Therefore, a novel type of thermal insulation coal gangue concrete using coal gangue is proposed [19]. Besides coal gangue, glazed hollow bead (GHB), nano-silica (NS), and ultra-fine slag (UFS) are also used to improve the performance of the novel concrete. As shown in Figure 12, increasing the CG content of the concrete would significantly reduce its thermal conductivity coefficient, even though the GHB content marks the maximum influence on the thermal conductivity coefficient of the concrete, while the NS and UFS content show subtle effects.

## 6. Application of Coal Gangue Concrete

### 6.1. Reinforced Coal Gangue Concrete

#### 6.1.1. Beam

**Figure 13:**
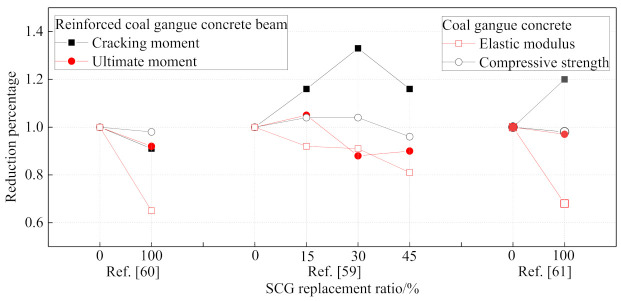
Flexural performance reduction of the reinforced coal gangue concrete beam.

In [59,60,61], the mechanical performance of SCG concrete and the flexural performance of reinforced coal gangue concrete beam are both studied. As shown in Figure 13, the reduction of the ultimate moment for reinforced coal gangue concrete beams is a little larger than that of the coal gangue concrete itself, while the cracking moment of reinforced coal gangue concrete seems unrelated to the coal gangue concrete replacement ratio, which may result from the various properties of coal gangue. In general, the ultimate moment reduction of reinforced coal gangue concrete beam is below 10%, which seems acceptable in practical engineering. Furthermore, [59,60,61] also indicate that the current design method in Chinese standards could be directly used to predict the flexural resistance of the reinforced coal gangue concrete beam by using the corresponding compressive strength of the coal gangue concrete.

**Figure 14:**
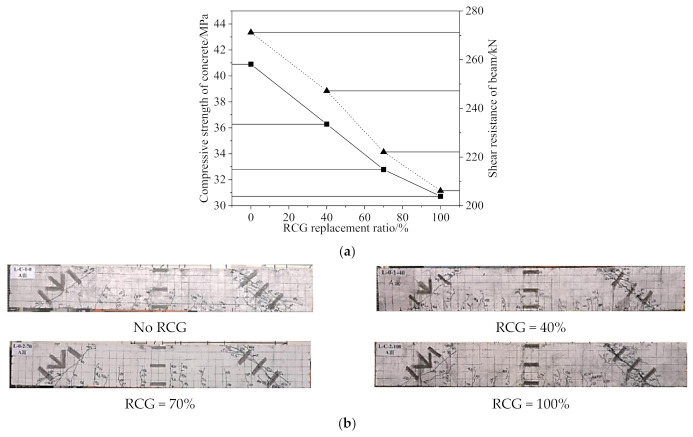
Shear performance of a reinforced coal gangue concrete beam [62]. (**a**) Shear resistance reduction; (**b**) Shear−compression failure patterns.

As shown in Figure 14 [62], the compressive strength reduction of coal gangue concrete is remarkable, which results in a remarkable reduction in the shear resistance of the reinforce coal gangue concrete beam. Regardless of the CG replacement ratio, the beams show a shear–compression failure pattern. The shear resistance of reinforced coal gangue concrete beams could also be predicted by the current design method using Chinese standards.

#### 6.1.2. Column

**Figure 15:**
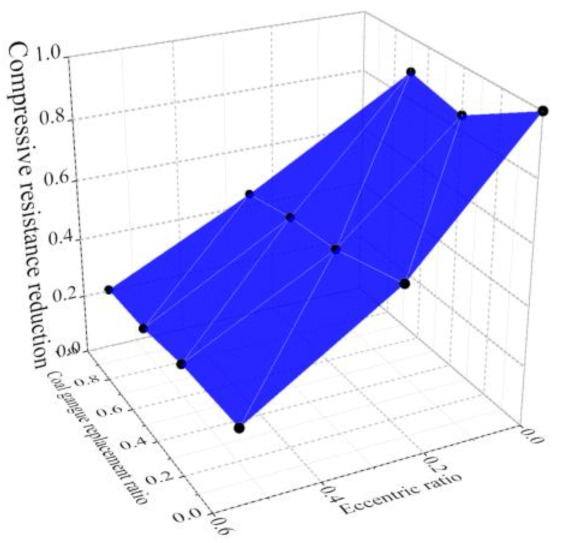
Compressive performance of reinforced coal gangue concrete columns.

The reinforced concrete columns using RCG show similar failure patterns to the normal reinforced concrete columns under axial and eccentric compression [63], except that the cracking load of the columns is reduced by increasing the CG replacement ratio. The effects of the eccentric ratio and CG replacement ratio on the resistance reduction of the columns are shown in Figure 15. The compressive resistance of reinforced coal gangue concrete columns could be predicted by the current design method in Chinese standards. In contrast, a large coal gangue replacement and axial compression ratio is not suggested in the columns under seismic loads [64].

#### 6.1.3. Wall

**Figure 16:**
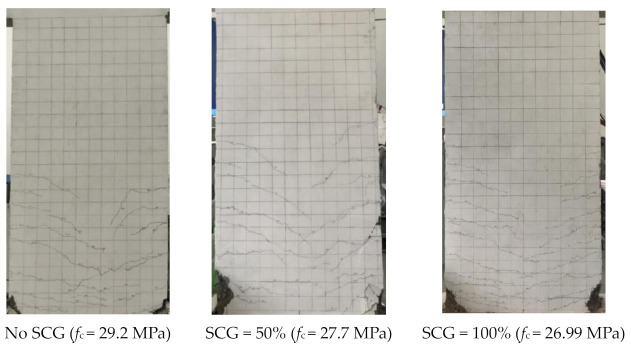
Failure patterns of coal gangue concrete wall [65].

As shown in Figure 16, the failure pattern of coal gangue concrete shear wall under cyclic loads is similar to that of a normal concrete shear wall, even though the failure of coal gangue concrete shear wall tends to be slightly more severe when raising the CG replacement ratio [65,66]. About a 10% reduction in the horizontal resistance of the wall was caused when using a 100% SCG replacement ratio, while the ultimate horizontal displacement of the wall was reduced by 3%. By considering the degradation of shear resistance under cyclic loads, namely a 30% reduction, the current design method in Chinese standards was modified for the shear resistance of coal gangue concrete wall [67].

### 6.2. Coal Gangue Concrete Filled Steel Tube

As mentioned above, the mechanical and durability performance of concrete is degraded when using coal gangue concrete. Therefore, concrete filled steel tubes (CFSTs) are an applicative configuration for using coal gangue concrete, as the external tube not only enhances the mechanical behavior of the internal core coal gangue concrete, but also protects it from the effect of severe environment [68,69,70].

**Figure 17:**
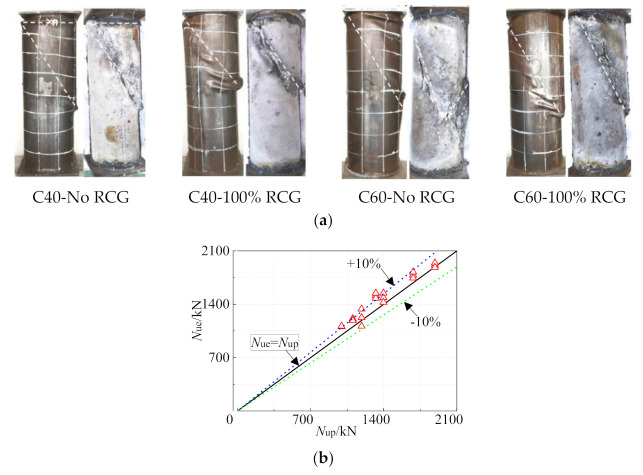
Compressive performance of coal gangue concrete filled steel tubes [72]. (**a**) Failure pattern; (**b**) Axial strength prediction.

Similar to reinforced coal gangue concrete stubs, the failure pattern of CFST stubs with coal gangue is also barely influenced by the CG replacement ratio, regardless of the concrete grade, as shown in Figure 17a. The axial strength of CFST with coal gangue is linearly related to the RCG replacement ratio [71]. Under a 100% replacement ratio, the axial capacity of CFST with coal gangue is reduced by 38%, while the compressive strength of coal gangue concrete is reduced by 50%. The current design method in Chinese standard GB50936-2014 for normal CFST was modified regarding the effect of CG replacement ratio, as follows [72]. The corresponding error of the modified method was in the range of ±10%. Even though this semi-empirical prediction method is proposed here for the first time, it is only valid for the CG from specific origins, as with the above-mentioned formulas regarding CG concrete.

(5) $$N_{up}^{\mathrm{CGCFST}}(r)=(A_{c}+A_{s})(1.212+(\frac{0.176f_{y}^{}}{213}+0.974)\;\xi +(\frac{-0.104k_{sr}^{\mathrm{CGC}}f_{c}}{14.4}+0.031)\;\xi^{2})k_{sr}^{\mathrm{CGC}}f_{c}$$

where $k_{sr}^{\mathrm{CGC}}$ is the strength reduction factor of coal gangue concrete, $k_{sr}^{\mathrm{CGC}}=1-0.909r_{\mathrm{CG}}+0.425r_{\mathrm{CG}}{}^{2}$.

### 6.3. Steel−Coal Gangue Concrete Composite Structure

#### 6.3.1. Wall

**Figure 18:**
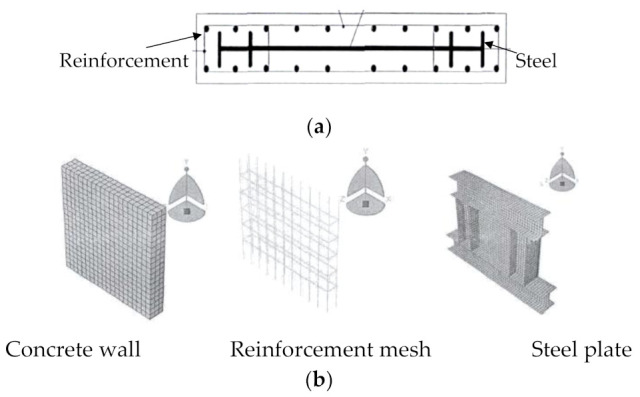
Coal gangue concrete shear wall with an internal steel plate [73,74]. (**a**) Profile of the tested composite wall; (**b**) Finite element model of the composite wall.

Some researchers have proposed setting an internal steel plate into reinforced coal gangue concrete walls to enhance their seismic capacity [73,74], as shown in Figure 18. Even though this configuration is supposed to be beneficial, and both experimental and numerical studies have been conducted in [73,74], the effect of the CG replacement ratio has not been represented in these studies, which mostly focus on the effect of the internal steel plate, rather than the coal gangue concrete. In addition, the simplified theoretical prediction for the shear resistance and the seismic model of this coal gangue concrete shear wall with internal steel plate is also not presented.

#### 6.3.2. Slab

**Figure 19:**
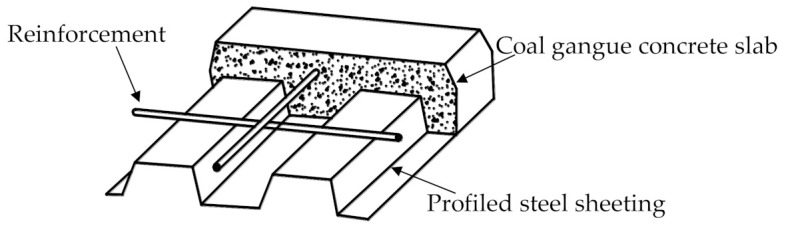
Composite slab with coal gangue concrete and profiled steel sheeting.

As a light-weight aggregate (bulk density less than 1200 kg/m^3^ [75]), coal gangue is also preferable in concrete slabs, especially with profiled steel sheeting, namely composite slabs, as shown in Figure 19. However, the performance of the slab with coal gangue concrete and profiled steel sheeting is still limited. Some preliminary studies confirm the feasibility of using coal gangue concrete in composite slab with profiled steel sheeting, even though the deflection of the slab with coal gangue concrete is about 10% larger than that with normal concrete [76,77].

## 7. Discussion and Research Gap

Based on the above analysis, generally, the properties of coal gangue coarse aggregates are feasible for substituting natural coarse aggregates in the preparation of green concrete. The mechanical performance, durability performance, and even the thermal characteristic of the coal gangue coarse aggregate concrete have been investigated. Even though the properties of coal gangue vary according to origins, the usage of coal gangue coarse aggregate would decrease the mechanical and durability performance, but improve the thermal characteristic of the concrete. The mechanical behaviour of coal gangue concrete is generally acceptable in practical applications, while its durability is relatively poor. The proposed stress−strain models of coal gangue concrete could be used in the numerical and theoretical prediction for its mechanical performance [77,78]. However, these models are empirically-based, and are highly related to the properties of the local coal gangue.

In contrast, the application of coal gangue coarse concrete in structural members is relatively limited. The mechanical performance of reinforced concrete, CFST, and steel−concrete composite structures with coal gangue concrete have been investigated considering the structural members of beams, columns, and walls. In particular, most of the formulas for the load-bearing capacity prediction of these members do not even consider the influence of the CG replacement ratio [79,80]. 

It is worthy of attention that CFST is an applicative configuration for using coal gangue concrete, even without a thorough study on its durability performance, since the external tube could protect core coal gangue concrete from the effect of severe environments. The prediction method for the axial resistance of CFST with coal gangue by directly considering the influence of the CG replacement ratio is relatively accurate and simple for practical application. 

It has been found that structural concrete-based coal gangue has several issues that, need to be resolved before its usage in the structural engineering. The following research gaps have been identified.

(1) The physical properties of RCG and SCG should be investigated comparatively in particular, since they are quite crucial to the properties of coal gangue concrete. In other words, the physical change of coal gangue as a coarse aggregate in concrete mixture before and after spontaneous/artificial combustion should be compared and identified quantitatively.
(2) Even though the main content in the chemical composition of RCG and SCG is actually stable, heavy metal and organic matter leaching of RCG and SCG should be studied comparatively. More specific permission regarding heavy metal and organic matter leaching in coal gangue should be issued by governments to avoid posing environmental, health, and safety concerns.
(3) The workability of coal gangue concrete significantly varies from the gangue origins, which makes the improvement method ineffective in some cases. This phenomenon may be solved if the physical properties of coal gangue are assessed more accurately. A proper index to assess the workability of coal gangue concrete for practical use should also be proposed based on more experimental data.
(4) Even though the degradation effect of coal gangue aggregate on the mechanical properties of coal gangue concrete is obvious, the empirical stress−strain model should be validated against more data. In particular, the elastic modulus and tensile strength of coal gangue concrete have not been described through theoretical models, not to mention bending and creep performance and damage accumulation process.
(5) The studies on the durability performance of coal gangue concrete are directly related to the properties of the coal gangue aggregate. Therefore, the specific properties and replacement ratio of coal gangue should be considered in the prediction formulas regarding the durability performance of coal gangue concrete. More tests on the durability performance of coal gangue concrete are still needed, such as the drying and watering cycle, high temperature, abrasion resistance, and sulfate resistance.
(6) Besides the experimental and theoretical studies on the structural members with coal gangue concrete are rather limited, the durability performance of those has never been studied. Specifically, the interface bonding behavior between the steel component and coal gangue coarse aggregate concrete under the severe environment and long-term loading should be studied in detail. More design methods from formal standards should be modified by considering the properties of coal gangue for practical applications.

## 8. Conclusions

This paper has reviewed the utilization of coal gangue (CG) in green concrete as a coarse aggregate. The mechanical and durability performance of coal gangue concrete and the corresponding structural members have been analyzed from the reported literature. The main conclusions are as follows:

(1) The physical and chemical properties of rock and spontaneous-combustion coal gangue are generally suitable for being used as a coarse aggregate in green concrete, even though these properties vary from the origin. The short-term heavy metal leachability of coal gangue is also relatively weak. However, more specific permission regarding heavy metal and organic matter leaching in coal gangue should be issued by governments to avoid posing environmental, health, and safety concerns.
(2) Coal gangue concrete is not recommended to be used in subsurface structures, since its water absorption law would be changed by increasing the CG replacement ratio. The slump of coal gangue concrete with a 100% CG content would be reduced by about 10–20%. Adding fly ash, superplasticizer, and additional water are effective and simple ways to enhance the workability of coal gangue concrete. A proper index to assess the workability of coal gangue concrete for practical use should also be proposed based on more experimental data.
(3) The mechanical performance, including compressive strength, elastic modulus, and splitting tensile strength of coal gangue concrete is degraded by raising the CG replacement ratio. This degradation caused by the porous structure of CG varies significantly from the CG origin. Over-low and -high concrete grades are not suggested to be used as coal gangue aggregates, unless extra admixture or specific methods are used. The models for describing the elastic modulus, tensile strength, bending strength, and creep performance of coal gangue concrete are still needed for practical applications.
(4) The porous structure of coal gangue provides more transmission channels for air and liquid in concrete. Therefore, the influence of CG on the durability of coal gangue concrete is more remarkable than on its mechanical performance, which could be reduced by adding super-fine admixture and calcination. In contrast, the porous structure of CG is beneficial in the preparation of thermal insulation concrete. The specific properties and replacement ratio of coal gangue should be considered in the prediction formulas regarding the durability performance of coal gangue concrete.
(5) The application of coal gangue concrete in structural members is still limited. Even though the static and seismic behavior of some structural members using coal gangue concrete has been investigated, the durability of these structural members has never been reported. Specifically, the interface bonding behavior between steel component and coal gangue coarse aggregate concrete under the severe environment and long-term loading should be studied in detail. Among them, concrete filled steel tubes are a preferable configuration for using coal gangue concrete, regarding both the mechanical and durability performance.

## Figures and Tables

**Table 1:** Physical properties of coal gangue aggregate from different origins in China.

Type	Region of China	Province	Apparent Density (kg/m^3^)	Bulk Density (kg/m^3^)	Water Absorption (%)	Crushing Value (%)	Void Ratio (%)	Ref.
SCG	Northeast	Heilongjiang	2588	1289	**3.8**	18.9	50.2	[13]
		Liaoning	2497	-	9.80	25.2	-	[12]
		Liaoning	2630	1550	8.5	16.6	**22.0**	[14]
		Liaoning	**2276**	1220	7.55	21.2	52.8	[15]
	South	Anhui	2630	**976**	9.2	16.6	57.9	[16]
		Jiangsu	2624	1430	4.88	24.4	-	[17]
RCG	Northeast	Liaoning	2653	1489	3.15	**9.9**	49.5	[15]
	North	Shanxi	**2083**	1130	4.7	18.7	-	[18]
		Shanxi	2689	1201	**11.4**	18.1	-	[19]
		Beijing	2640	-	1.8	22.6	-	[20]
		Henan	2510	1320	1.7	19.1	47.5	[21]
		Henan	2540	1264	3.9	17.9	51.5	[22]
	Northwest	Shaanxi	**2090**	-	4.92	19.0	-	[23]
	South	Jiangsu	2712	-	1.7	16.8	-	[24]
		Jiangsu	2620	1612	3.9	18.6		[17]

**Table 2:** Mineralogical composition of coal gangue in various countries/% [26].

Minerals	Czechoslovakia	Germany	Spain	Britain	USSR	China
Illite	10–45	41–66	20–60	10–31	5–30	10–30
Kaolinite	24–45	4–25	3–30	10–40	1–60	10–67
Chlorite	0–15	1–3	0–7	2–7	--	2–11
Quartz	10–50	13–27	5–57	15–25	--	15–35
Iron ore	0–25	0.5–5	--	2-10	0.2–8	2–10
Organic matters	0–25	5–10	4–30	5–25	8–40	5–25

**Table 3:** Chemical composition of CG (mass fraction)/%.

Type	Region	Province	SiO_2_	Al_2_O_3_	Fe_2_O_3_	CaO	MgO	Na_2_O	K_2_O	TiO_2_	P_2_O_5_	SO_3_	Loss	Ref.
RCG	Northeast of China	Heilongjiang	61.0	23.6	6.70	1.18	0.52	-	-	-	-	-	2.50	[11]
		Heilongjiang	**34.1**	26.0	0.49	0.67	0.61	-	0.16	-	-	0.28	**32.8**	[27]
		Liaoning	50.3	26.3	6.11	7.74	2.00	1.10	3.28	1.13	0.15	0.93	-	[15]
	North of China	Beijing	49.9	24.4	6.42	0.82	1.59	1.46	2.06	0.88	-	0.12	11.8	[3]
		Shandong	48.8	19.0	4.47	2.03	2.29	1.43	0.19	-	-	-	16.8	[3]
		Henan	59.9	20.7	6.70	2.00	1.80	0.65	2.40	-	-	1.53	-	[7]
		Shanxi	35.1	16.8	**27.3**	3.82	1.60	-	-	-	-	3.00	0.83	[19]
		Hebei	48.3	23.1	4.30	4.10	1.70	0.10	1.50	0.80	0.10	1.00	14.7	[28]
	Northwest of China	Shaanxi	49.5	33.3	7.60	6.09	0.97	0.52	0.94	0.83	-	-	-	[23]
	South of China	Jiangsu	55.5	18.2	5.42	3.38	1.23	0.64	1.67	-	-	0.64	13.3	[3]
		Jiangsu	59. 8	29.4	1.44	0.68	0.51	0.08	1.76	-	-	-	-	[8]
	Poland	-	58.9	20.5	6.63	0.35	1.93	0.54	3.19	1.05	0.06	0.17	5.50	[5]
	Italy	No. 1	43.7	21.4	5.57	0.89	0.77	0.11	0.16	1.05	0.16	1.02	25.2	[29]
		No. 2	46.8	17.2	7.67	7.60	0.99	0.15	2.40	0.78	0.16	0.34	15.8	
		No. 3	57.2	18.7	6.25	1.86	1.42	0.46	3.59	0.95	0.14	0.08	9.28	
SCG	Northeast of China	Liaoning	55.6	21.0	6.57	3.65	2.50	1.90	4.10	0.84	0.24	0.82	-	[15]
	North of China	Shanxi	56.6	36.8	1.95	0.62	0.22	0.42	-	2.10	-	-	-	[9]
	Northwest of China	Shaanxi	55.2	31.1	2.94	1.31	0.75	0.12	1.13	1.12	0.07	-	5.94	[30]
	Italy	No. 1	56.4	26.3	6.42	1.06	1.07	4.02	0.17	1.21	0.20	0.65	2.38	[29]
		No. 2	52.3	19.5	8.35	8.41	1.21	2.65	0.20	0.85	0.17	0.49	5.61	
		No. 3	61.9	20.2	6.77	2.00	1.57	3.78	0.53	1.01	0.15	0.11	1.81	

**Table 4:** Content of heavy metals in coal gangue/mg kg^−^^1^.

Type	Region	Province	As	Ba	Cd	Co	Cr	Cu	Hg	Mo	Ni	Pb	Sn	Zn	Ref.
RCG	China	Jiangsu	27.3	-	0.58	-	42.3	24.3	0.24	-	20.5	4.32	-	39.5	[31]
SCG	Italy	No. 1	-	-	-	-	**190**	-	-	-	56	11	-	27	[29]
		No. 2	-	-	-	-	**228**	-	-	-	43	5	-	46	
		No. 3	-	-	-	-	**308**	-	-	-	57	10	-	37	
RCG	Poland	-	<2	84.5	2.9	14.6	61.9	48.3	0.04	<2	41.2	20	3	89.5	[5]
Permission content in Poland			20	200	4	20	150	150	2	10	100	100	20	300	[5]
